# Feasibility of minimum intervention oral healthcare delivery for individuals with dental phobia

**DOI:** 10.1186/s12903-023-03095-8

**Published:** 2023-07-31

**Authors:** Ellie Heidari, Avijit Banerjee, Jonathon Tim Newton

**Affiliations:** 1 Faculty of Dentistry, Oral & Craniofacial Sciences, King’s College London, Guy’s Dental Hospital, Great Maze Pond, London, SE1 9RT UK; 2 Professor of Psychology as applied to Dentistry/Honorary Consultant/Dean of Research Governance, Ethics and Integrity, Faculty of Dentistry, Oral & Craniofacial Sciences, King’s College London, London, UK

**Keywords:** Minimally invasive, Dentistry, Minimum intervention, Anxiety, Phobia, Prevention

## Abstract

**Background:**

People with dental phobia often present with more active dental caries and fewer teeth. Minimally Intervention oral Healthcare offers a possible solution to address the high care needs of this group. The aim was to determine this patient group’s eligibility and willingness to participate and the effect of MIOC, compared to treatment as usual (TAU), on their oral health outcomes for planning a future randomised controlled trial (RCT). Minimum intervention oral healthcare (MIOC) comprises of four interlinked domains. In the first domain, we identified and diagnosed the disease status and participants’ anxiety status (≥ 19 MDAS). In the second domain, an individualised prevention-based personalised care plan was designed. During this process, patients with dental phobia were exposed to the dental environment in a stepped manner (‘graded exposure’) and had their urgent care provided with conscious sedation. In the 3^rd^ domain, we took a minimally invasive operative approach to restore teeth while preserving tooth substance and limiting the use of fear-provoking stimuli (e.g., rotary instruments) when possible. At the review and the recall appointment(s) (4^th^ domain), the patients’ oral health care behaviours, disease risk/susceptibility and fear levels were re-assessed.

**Methods:**

This two-arm randomised feasibility trial (*N* = 44) allocated participants to the experimental arm (MIOC) or the control arm (treatment as usual [TAU]). The primary outcomes were the eligibility and willingness to participate and feasibility to conduct a trial of MIOC for people with dental phobia. The secondary outcomes were oral health status, oral health related quality of life and care completion. A written and verbal consent for participation and dental care provision were obtained.

**Results:**

Forty-four people diagnosed with dental phobia were allocated randomly to the two study arms. At the six-month recall after completed care, the outcome of each study arm was assessed. It was feasible to conduct a clinical trial (eligibility rate [56%], completion rate [81%], declined to participate [12%]). The intervention group showed improvements in all health care outcomes, and oral health related quality of life.

**Conclusion:**

A clinical trial of MIOC vs TAU in people with dental phobia is feasible. Preliminary findings suggest that patients in the MIOC arm are more likely to successfully complete their course of treatment. The study was ‘retrospectively registered’ on 02/05/2018 (ISRCT15294714) with the International Standard Randomised Controlled Trial (ISRCT).

## Background

Dental phobia has been defined “as a persistent fear of situation disproportional to the actual danger posed to the affected person where many will go to great lengths to avoid it. If the feared object or situation cannot be avoided entirely, the affected person will endure it with marked distress and significant interference in social or occupational activities” [1]. Its negative impact on oral health related quality of life (OHR QoL) has been previously documented [2].

There are multiple contributing reasons to poor oral health which is mostly preventable. These include socioeconomic status (being in routine occupations and with lower educational attainment), psychosocial factors (anxiety and fear) and proximal determinants that include biological (inflammation) [3] and behavioural influences (OHR behaviours such as diet, tobacco use, hygiene practices). In a study, there were more people in the dental phobia group who brushed their teeth intermittently (never or occasionally) and did not use a variety of oral hygiene products (mouthwash, floss sugar-free gum, interspace cleaning, and use of electric toothbrush) than the non-phobic groups [2].

Other oral health-related contributing factors can be poor patient treatment choices [4] and self-rated oral health, history of oral pain [5], irregular attendance [6], lack of knowledge about preventive measures and attitudes towards oral health. The lack of dental services with adjunctive behavioural management elements such as cognitive behavioural therapy [CBT]) and conscious sedation (CS) techniques for this group might be considered to be other contributing factors [7].

Many phobic people (798 [58.5%]) even with an awareness of their dental needs, will wait to seek care and will only attend with painful (infected) symptomatic teeth that exhibit longstanding untreated dental caries [2]. Regular attendance not only leads to maintenance of good oral health but also will prevent active caries progression to ultimately, unrestorable teeth that will require extraction leading to tooth loss and poor OHR QoL.

The minimum intervention oral healthcare (MIOC) is the underlying principle of best clinical practice as it takes a holistic approach to provide personalised oral healthcare. The pathway’s first domain involves identification of patients’ problems (detection, risk/ susceptibility assessment of oral diseases), to enable diagnosis and patient-focused care planning. The 2^nd^ domain entails of prevention (primary, secondary and tertiary prevention of lesions) and control of the disease process detailed in an individualised programme for the patients. The 3^rd^ domain encourages minimally invasive operative management of oral health diseases (caries and periodontal disease) to manage its impact (fractured/broken down teeth, pulp and periodontal pathology) on oral health. The 4^th^ domain is the recall and review assessment of the provided treatment and patient’s behaviours. In this process the identification of the patients’ susceptibility for developing disease is re-assessed to contribute to the new developed care plan.

In this programme (MIOC pathway for people with dental phobia), the level of support for prevention and control of disease in MIOC pathway is suitable for this patient group, in particular for caries element because of its chronic and accumulative nature [8]. The control of disease in the initial and non-cavitated carious lesions and the non-operative management of it with an attempt to arrest the caries process with emphasis on remineralisation using fluoride varnish applications [8, 9] can have a long term impact on the patient with an increased retention of teeth hence the likelihood improvement of the patients’ OHR QoL. The suggested improved care can reduce overutilization of emergency dental care services [10] as the programme provides them with tools for self-care and utilisation of preventive oral health services. The programme, during the 2^nd^ and 3^rd^ MIOC domains, offered conscious sedation (CS) for dental treatment to the patients to enable care provision whilst been supported to overcome their dental phobia by offering CBT services in SSCD [11] upon request by patients and at the recall appointment.

The learnt skills to combat dental phobia (for example the learnt relaxation techniques) can be used when patients are exposed to trigger factors (e.g., local anaesthetics). In the Minimal Invasive Dentistry (MID) (3^rd^ domain) approach, the use of dental drills (a common trigger factor for dental anxiety) is minimised for dental procedures as removal of the cavitated carious tissue in the active lesions is done selectively (with only the soft infected dentine being removed). During these procedures, there is a chance to maintain pulp sensibility and preserve the dentine-pulp complex and the functional tooth structure state in the long term [8].

The gained knowledge (e.g., dental phobic people’s experiences of dental services) from previous studies [2, 7, 12] led to shaping the aims for this study. The feasibility assessment of conducting a randomised controlled trial (RCT) by collecting data about recruitment, eligibility, acceptability of MIOC pathway for this patient group and completion rates was investigated. The other objectives were to determine feasibility and preliminary effect size for MIOC vs Treatment as Usual (TAU), participants’ views and the impact of MIOC on oral health outcomes (by collecting clinical data) and OHR QoL.

## Methods

### Trial design

**Fig. 1:**
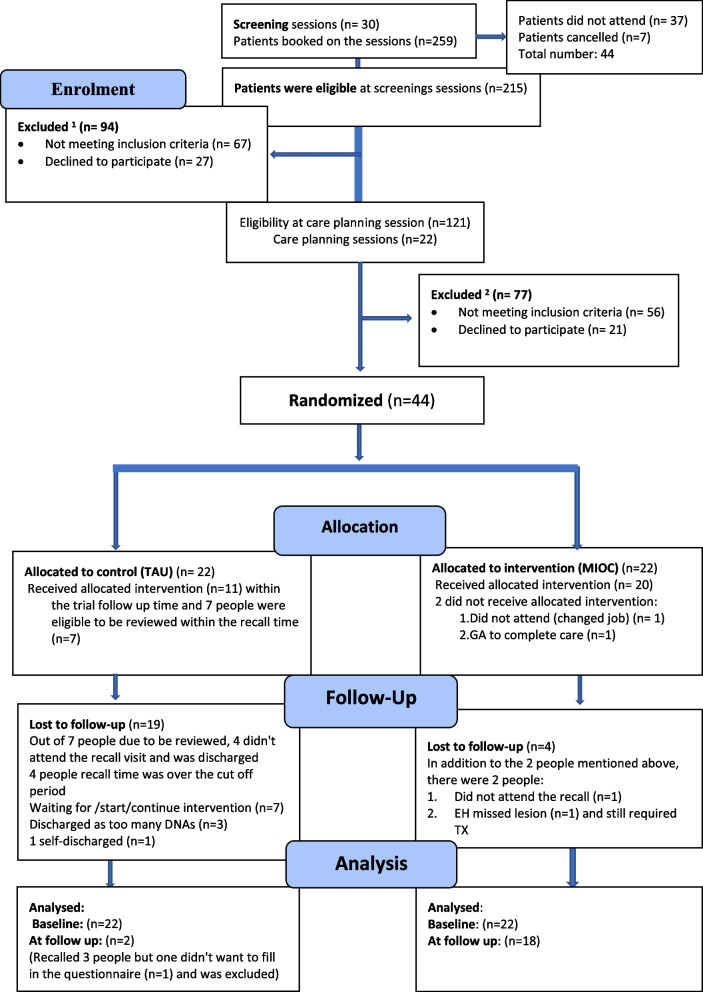
Flow diagram for the feasibility study according to CONSORT 2010

A two-arm feasibility trial (Fig. 1) comparing a MIOC pathway for people with dental phobia versus treatment as usual (TAU), was conducted with 44 patients. A sample size recommended previously for feasibility studies [13], was used in this secondary care setting. The eight-month recruitment period ran between September 2017 and April 2018. The end date for treatment completion (follow-up appointments) was February 2019.

The following three feasibility outcomes: the proportion of patients presenting for treatment who met the eligibility criteria (Table 1); the proportion of eligible patients who were willing to participate, and the proportion of participants who completed the trial (Fig. 1), were investigated in this study. In addition, the study sought to determine a preliminary estimate of the following potential outcome variables:

- Dentist adherence to intervention.
- Patient adherence with behaviour required by intervention and completion of personalised care plans.
- Proportion of participants captured at recall and patient satisfaction. Other outcome measures were estimates of means and standard deviations.

**Table 1:** The inclusion and exclusion criteria for the feasibility trial

Heading	Details
***Inclusion Criteria***	Have been accepted for dental care at SSCD
	Patients who have agreed to participate and signed the consent form
	Are 18 years old and above
	Have a diagnosis of dental phobia with Modified Dental Anxiety Score (MDAS) score > = 19
	The test for Depression and General Anxiety was measured by HADS: the participants will have no comorbid psychiatric conditions (when HADS is A < 10; HADS D < 10)
***Exclusion criteria***	Individuals with learning difficulty, difficulty in communicating or who are unable to give informed consent
	Potential participants who cannot understand, read and write English will be excluded as ability to communicate clearly is essential for this study

Approval was obtained from East of Scotland Research Ethics Service (EoSRES) and Health Research Authority (REC:17/ES/0067). The study was also registered with the International Standard Randomised Controlled Trial (ISRCTN15294714) with a link to the protocol. All methods were carried out in accordance with relevant guidelines and regulations. Informed consent was obtained from all participants for all forms of personally identifiable data including clinical, and biometric data.

### Setting and location

The department of Sedation and Special Care Dentistry (SSCD) is based in Guy's Dental Hospital as part of Faculty of Dentistry, Oral & Craniofacial Sciences, King’s College London (KCL) and Guy's and St Thomas' Hospitals Foundation Trust (GSTT). Patients are referred from primary care for treatment by specialists when care cannot be carried out in a primary care setting.

### Participants

The principal investigator (EH) approached adults with dental anxiety and phobia (according to the referral forms and SSCD acceptance criteria) that had been accepted for care at SSCD in the screening sessions (T); (Table 2). While no classically vulnerable groups (e.g. children, those are unable to consent) were recruited, all patients who met the entry criteria (Table 1) were approached to participate to the study.

**Table 2:** A simplified version of the events’ matrix

	**Visit 1 (T1)**	**Visit 2 (T2) (care planning session)**	**Visit 3 (T3)**	**Recall appointment after 6 months**
Patient Information Sheet (PIS) was given	X			
All patients who were accepted for dental care had radiograph examination according to their needs. This is a standard process and was not dependent on participation in this study	X			
Consent- a signed copy was given to the patient		X		
Part A questionnaire: Screening data: HADS ( A and D > 10) and MDAS: (19 or above)		X		
If eligible: Letter to GMP/GDP		X		
If not eligible and high scores of HADS a letter to GMP		X		
If eligible: Randomisation In the intervention group: EH provided care for all the patients In the TAU group: Patients were allocated to various members of staff for care planning sessions		X		
Oral health needs and caries risk assessment: In this part, the radiographs findings (radiographs that was taken when patients attended the department at visit 1) complemented the oral finings when an oral health examination took place In both groups, a personalised prevention program was offered as a referral to the SSCD dental therapist and hygienist at the same visit was introduced on the 08 November 2017 **In the intervention group:** **There were discussions about:** • MIOC and MID principles • Patients’ expectations/needs/wishes • Provisional nature of the provided care plan • Behavioural management and CS techniques **Clinical examinations (if possible):** • Tooth sensibility (vitality) test: **Measures that were used:** • A clear visual-tactile evaluation when possible: Decayed, Missing, Filled Teeth/Surfaces (DMFT/DMFS) scores was calculated (according to ADHS 2009 criteria) • Periodontal status (2009 ADHS criteria) • Plaque score • Basic Periodontal Examination (BPE) when possible **In the TAU group:** **There were discussions about:** • Patients’ expectations • Provisional nature of the provided care plan • Behavioural management and CS techniques **Clinical examinations (if possible):** • Decayed, Missing, Filled Teeth/Surfaces (DMFT/DMFS) • Basic Periodontal Examination (BPE) (introduced on the 08 November 2017) at the same visit In both groups, at the care planning sessions, a provisional care plan was discussed and agreed upon		X		
Treatment sessions: **In the intervention group:** EH provided care for ‘preventive oral health related’ and treatment sessions At each visit, EH gave OHI and followed up patients’ commitments to OH practises. EH used behavioural technique management (such as relaxation techniques by using controlled breathing) during these sessions **During the sedation:** 1^st^ sedation appointment scale and polish (Professional Mechanical Plaque Removal [PMPR]) as well as dental treatment • The restorative treatment protocol (MID): A. Started with teeth that presented with the deepest lesions first: partial caries removal (PCR) with rubber dam to protect the pulp-dentine complex B. Monitored initial carious lesions and poor prognosis teeth • BPE and minimum periodontal care **At the recovery, information to the patients:** • Reinforced the importance of OHI and discussed about MID and MIOC • What the continuation of the care plan would be (e.g., to continue to restore teeth with deepest lesions at the following visits) • Monitor prognosis of teeth In the TAU (the control arm), SSCD staff or diploma students in SSCD provided care for the patients The common procedures were: **Pre sedation:** • The care plan was dictated by previous care plan. At sedation appointment the care plan could also be influenced by the patient especially when presented with symptomatic teeth and the practitioner’s preference **During the sedation:** • 1^st^ sedation appointment: usually scale and polish or relieve of pain (e.g., extractions of appropriate teeth if the patient is in pain) • with exception by one dental therapist, the caries management was by complete excavation (the hardness of dentine is determining the completion of excavation) **Recovery:** • To be allocated to either a member of staff or a SSCD postgraduate student			X	
Both groups were seen by the dental therapists/dentists in the SSC department **1. The 'study questionnaire' had 3 parts:** **Part A:** demographic information and MDAS and HADS (screening questionnaires) that was given at the beginning of the study **Part B** of the questionnaire (with questions based on ADHS, 2009) (O’ Sullivan et al., 2011) documented OHR behaviours (such as toothbrushing) and QoL (measured by Oral Health Impact Profile [OHIP 14]) Whereas **part C** sought participants’ views ( in the adapted version of Treatment Evaluation Inventory [TEI]) (Newton & Sturmey, 2004) post completion of care (≥ 6 months) The entire questionnaire could take approximately 35 min to complete All 3 parts of the questionnaire (A, B and C) and an oral health assessment was conducted by a member of SSCD staff **2. For both arms, the clinical measurements** • Full charting (for EH to calculate DMFT/DMFS) • BPE • Plaque score (used disclosing tablets) If treatment needed, a staff would review the provisional care plan and offer CBT If no further treatment was required, the patient was referred back to the GDP in both arms • In the intervention group, an additional letter that detailed the MID/MIOC principles and suggested a recall time based on the individual’s risk/susceptibility (e.g., monitoring the integrity of the sealants) and management of the future oral health diseases was sent to the patient’s GDP. When applicable, the letter also suggested to review the provisional care plan considering rehabilitation and replacement of missing teeth The questionnaire can be sent by request.				X

Once patients had been screened for suitability (MDAS ≥ 19 and HADS questionnaire), they were invited to participate to the study. In T1, the study was explained and a Patient Information Sheet (containing the study details) was distributed, discussed and given to the patient to take home to consider. Once the patients accepted to participate (first stage of consent), upon their return for care planning (T2), the second stage of consent was obtained, and the eligibility criteria assessed (Table 1) by using a prospective questionnaire (part A).

### Randomisations

Patients were allocated to condition at random, following a simple random allocation sequence created independently of the treating clinician. The allocation sequence was concealed in an opaque envelop, opened after the participant consented.

### Intervention (T3)

The intervention group was treated according to the MIOC patient management pathway [14] based on the caries risk/susceptibility assessment (after identification of risk factors such as oral hygiene [OH] procedures, medical problems, diet, lesion location, etc.). A personalised preventive programme (e.g., OH instruction [OHI], diet advice and fluoride varnish) was introduced. The programme was delivered on multiple occasions based on the patients’ susceptibility risk / behaviours (e.g., sugar intake and OH regime) before the start of the treatment session by EH in the intervention (MIOC) group. A complete course of dental treatment according to MIOC protocols and behavioural management was provided.

### Description of treatment as usual arm (TAU)

Participants in the standard (control, TAU) care arm were offered conventional treatment. Upon completion of dental care, at the recall appointment that marked the end of the study, participants’ oral health outcome was documented by SSCD staff (Table 2). Failing to book or attend the recall appointment marked the end of study. Table 3 outlines the data that was collated.

**Table 3:** The trial’s data collection information

Heading	The collected data
The trial’s recruitment related issues and the suggested MIOC treatment provision	Number of **eligible patients** attending
	Percentage of **eligible patients recruited**
	**Dentist’s adherence to intervention** (assessed by independent review of case notes) defined as proportion adhering to specific behaviours within each intervention
	**Patient behavioural adherence** (assessed by self-report data from Part B questionnaire at the follow-up appointment)
	Proportion of participants **captured at follow-up**
The treatment outcomes and study participants’ views	**Treatment Evaluation Inventory (TEI)** (follow-up only, secondary outcome)
	Other measures:
	1. **DMFS** (baseline and follow-up)
	2. **Plaque score** (baseline and follow-up)
	3. **Basic Periodontal Examination Score** (baseline and follow-up
	4. **OHIP 14 score** (baseline and follow-up)

### Validation of the intervention

In order to validate the independent variable (testing if MIOC was delivered as per protocol design), one of the authors (AB) reviewed 11 randomised patients’ records to assess if treatment was provided per feasibility and MIOC protocol.

### Measures

Information was collected (Table 3) on the feasibility of conducting the trial (feasibility data); screening information from potential participants (screening measures) and demographic characteristics of participants and the trial outcomes.

### Screening measures

Participants completed a questionnaire set comprising Demographic data, Modified Dental Anxiety Score (MDAS) and Hospital Anxiety & Depression Scale (HADS) information. MDAS consists of five-items related to the dentist’s waiting room, tooth drilling and scaling in addition to local anaesthetic injection. The 5-point scale responses can vary from: Not Anxious (score of 1) to Extremely Anxious (score of 5). The sum can produce a total score ranging from 5 to 25 with cut-off value of 19 (score ^3^19) and above indicating dental phobia [15].

The general anxiety and depression were measured using HADS, a 14-item questionnaire that produces two scales, one for anxiety (HADS–A) and one for depression (HADS–D), with scores of ^3^ 11 a definitive case of psychological distress [16].

### Measures for eligible patients

The eligible participants filled in the questionnaire (part A and part B). Part B of the questionnaire (with questions based on ADHS, 2009) [17] documented oral health related behaviours (such as toothbrushing) and the subjective perception about oral health and wellbeing which was measured using QoL (measured by Oral Health Impact Profile [OHIP 14]) at baseline and post care completion. OHIP-14 is a subset of items from the OHIP-49 [18]. The dimensions are functional limitation, physical pain, psychological discomfort, physical disability, psychological disability, social disability and handicap.

At the recall appointment, in the post completion of care (≥ 6 months), in addition to part A of the questionnaire (patients’ demographic, MDAS and HADS), part B (patients’ OHR behaviours based on ADHS, 2009 and adherence with behaviour modification required by MIOC intervention) and part C (we sought participants’ views in the adapted version of Treatment Evaluation Inventory [TEI]) by Newton & Sturmey 2004. The questionnaire was disturbed to all feasibility study’s participants (Table 2).

The TEI measures acceptability of treatment interventions and has a 19-item questionnaire with answers on a seven-point Likert scale with higher scores indicating more perceived acceptability [19].

### Outcome measures (baseline and completed care)

The clinical data compromised of collecting data on total number of teeth present, DMFS, BPE and plaque scores based on the UK ADHS 2009 methodology [17].

### Statistics

For outcome measures, the statistical analysis consisted of descriptive statistics (means, proportions, standard deviations [SD]). No changes to the trial protocol or trial measures were made after the commencement of the trial.

## Results

Figure 1 outlines the proportion of patients presenting for treatment who met the eligibility criteria, the eligible patients who were willing to participate and the number of participants who completed the trial. A total of 259 people were invited to attend for a conscious sedation (CS) screening appointment. Forty-four (17%) people did not attend or cancelled the appointment (Fig. 1). The response rate was 87% (188) and a total of 92 (43%) eligible patients were recruited to the trial. All 44 participants consented for dental treatment provision with CS (intravenous sedation with midazolam) (Fig. 1).

In addition, the study sought to determine a preliminary estimate of the following potential outcome variables (e.g., patient satisfaction with care provided) (Table 4).

**Table 4:** The data form the trial’s data collection information

Heading	The collected data	
The trial’s recruitment related issues and the suggested MIOC treatment provision	Number of **eligible patients** attending at screening sessions	*n* = 215
	Number of **eligible patients recruited at care planning**	*n* = 121
	**Dentist’s adherence to intervention** (assessed by independent review of case notes) defined as proportion adhering to specific behaviours within each intervention	In all cases
	**Patient behavioural adherence** (assessed by self-report data from Part B questionnaire at the follow-up appointment)	See Table 5
	Number of participants **captured at follow-up**	TAU = 2 out of 22 MIOC = 18 out of 22
The treatment outcomes and study participants’ views	**Treatment Evaluation Inventory (TEI)** (follow-up only, secondary outcome)	Table 7
	Other measures	
	1. **DMFS** (baseline and follow-up)	Table 6
	2. **Plaque score** (baseline and follow-up)	
	3. **Basic Periodontal Examination Score** (baseline and follow-up	
	4. **OHIP 14 score** (baseline and follow-up)	Table 6

### Validation of the intervention

One of the authors (AB) reviewed independently a random sample of 11 participants case records in the intervention arm and was satisfied that dentist (EH) had adhered to the defined specific behaviours in the MIOC pathway.

### Patient’s demographic

Most of the respondents were female (32, 72%) and between the ages of 45 to 54 (13, 30%). The average age for completing full-time education was 16 and 18 in the experimental respective control arm at the baseline. Majority of the respondents (33, 75%) only attended when they had trouble with their teeth/dentures.

In MIOC, the patients’ adherence to OHR behaviour (follow the individual tailored prevention regime in 2^nd^ domain of MIOC) required by the intervention was assessed using the self-reported data from the questionnaire (Part B) at the recall appointment (Table 5). At the recall appointment, most participants in the intervention group (MIOC), used an electric toothbrush (11, 61% up from baseline levels of 9, 41%) followed by a mouthwash (9, 50% up from the baseline levels of 7, 32%),). They also increased their use of interspace brush (7, 39%, up from the zero figure at the baseline) and sugar-free chewing gum (7, 39% up from the baseline levels of 4, 18%) (Table 5).

**Table 5:** The demographic and oral health-related behaviours of study participants

		**Baseline**		**Recall**	
**Variable**		**MIOC 22 people (%)**	**TAU 22 people (%)**	**MIOC 18 people (%)**	**TAU 2 people (%)**
**In general, do you go to the dentist for**	A regular check-up?	4 (18)	1 (4)	1 (5)	0
	An occasional check-up?	3 (14)	3 (14)	3 (17)	0
	Or only when you're having trouble with your teeth/dentures?	15 (68)	18 (82)	14 (77)	2 (100)
**The last visit at which you visited a dentist – it does not include a visit to the dental hygienist-was?**	Within the last 6 months	13 (59)	15 (68)	6(33)	0
	Within the last 7–12 months	3 (14)	2 (9)	9 (50)	1 (50)
	More than 1, but less than 2 years ago	0	0	3 (17)	1 (50)
	More than 2, but less than 3 years ago	1 (4)	0	0	0
	More than 3, but less than 5 years ago	1 (4)	1 (4)	0	0
	More than 5, but less than 10 years ago	0	1 (4)	0	0
	More than 10 years ago	4 (18)	3 (14)		
**How often do you clean your teeth nowadays?**	More than twice a day	1 (4)	4 (18)	0	0
	Twice a day	15 (68)	13 (59)	18 (81)	2 (100)
	Once a day	5 (23)	1 (4)	0	0
	Less than once a day	1 (4)	4 (18)	0	0
	Never	0	0	0	0
**Do you use anything other than an ordinary (manual) toothbrush and toothpaste for dental hygiene purposes?**	Yes	14 (64)	14 (64)	18 (81)	1 (50)
	No	8 (36)	8 (36)	0	1 (50)
**If yes, what do you use? (you can circle more than one option here)**	Dental floss	5 (23)	4 (18)	4 (22)	1 (50)
	Interdens/toothpicks/wood sticks	1 (4)	1 (1)	2 (11)	0
	Mouthwash	7 (32)	10 (45)	9 (50)	0
	Interspace brush	0 (0)	2 (9)	7 (39)	0
	Electric toothbrush	9 (41)	6 (27)	11 (61)	1 (50)
	Denture cleaning solution	1 (4)	0	0	
	Sugar-free chewing gum	4 (18)	2 (9)	7 (39)	
	Something else. Please specify:				
	Total dental hygiene products used	1 (1) 28	25	40	2
**Do you usually have sugar in hot drinks like tea and coffee?**	Yes	11 (50)	10 (45)	8 (44)	0
	No	11 (50)	12 (54)	10 (55)	2 (100)
**How often, on average do you have fizzy drinks, fruit juice, or soft drinks like squash, excluding diet or sugar-free drinks?**	6 or more times a week	2 (9)	5 (23)	2 (11)	0
	3–5 times a week	4 (18)	3 (14)	5 (28)	0
	1–2 times a week	4 (18)	5 (23)	2 (11)	0
	Less than once a week	9 (41)	5 (23)	2 (11)	1 (50)
	Rarely or never	3 (14)	4 (18)	7 (39)	1 (50)
**How often, on average, do you eat sweets or chocolate?**	6 or more times a week	4 (18)	1 (4)	1 (5)	0
	3–5 times a week	5 (23)	5 (23)	4 (22)	0
	1–2 times a week	7 (32)	9 (41)	6 (33)	1 (50)
	Less than once a week	3 (14)	5 (23)	4 (22)	1 (50)
	Rarely or never	3 (14)	2 (9)	3 (17)	0
**How often, on average, do you eat a serving of cakes, biscuits, puddings or pastries?**	6 or more times a week	4 (18)	0	4 (22)	0
	3–5 times a week	5 (23)	6 (27)	2 (11)	1 (50)
	1–2 times a week	7 (32)	8 (36)	5 (28)	0
	Less than once a week	4 (18)	4 (18)	2 (11)	1 (50)
	Rarely or never	2 (9)	3 (14)	5 (28)	0
	No answer	0	1 (4)	0	0
**Have you ever been given advice or help from a dentist or a member of the dental team about the food and drinks you should be consuming?**	Yes	8 (36)	10 (45)	17 (94)	2 (100)
	No	14 (64)	12 (54)	1 (5)	0
**Cleaning?**	Yes	15 (68)	15 (68)	18 (100)	2 (100)
	No	7 (32)	7 (32)	0	0
**Have you ever smoked a cigarette, a cigar, or a pipe?**	Yes	13 (59)	16 (73)	13 (72)	1 (50)
	No	9 (41)	6 (27)	5 (27)	1 (50)
**And do you smoke cigarettes at all nowadays?**	Yes	10 (45)	8 (36)	6 (33)	0
	No	12 (54)	14 (64)	assume from the answer above 12 (67)	2 (100)

### Estimates of means and standard deviations for outcome measure

The presence of carious lesions in this study was classified as: “teeth with visual caries or cavitated caries or teeth that were so broken down, possibly with pulp involvement, that they were unrestorable. It included teeth that had restorations with caries associated with restorations and sealants (CARS) but did not include teeth that had restorations which were lost, broken or damaged but where there were no signs of caries” (ADHS criteria, 2009).

The periodontal pocket scores were measured from the upper sextants using a “type C” probe which has marks at 8.5 mm and 11.5 mm as well as at 3.5 mm and 5.5 mm. The worst buccal pocket score was entered in the excel sheet (ADHS criteria, 2009). The mean and standard deviations for the outcome measures are presented in Table 6. At the recall appointment, in the MIOC group the mean number of teeth and decayed teeth was reduced whilst the number of filled teeth had increased.

**Table 6:** The clinical data for the study participants

	**MIOC baseline**		**TAU baseline**		**MIOC recall (18 people)**		**TAU recall (2 people)**	
	**Mean**	**Standard deviation**	**Mean**	**Standard deviation**	**Mean**	**Standard deviation**	**Mean**	**Standard deviation**
**Total no. of teeth**	**26**	**4.5**	**25.5**	**6.3**	**24**	**4.2**	**24**	**0**
**No. of decayed teeth**	5	5,8	7	5.6	0.5	1.4	0	0
**No. of missing teeth**	6	4.5	5	3.3	7	4.0	8	0
**No. of filled teeth**	5	3.7	5	5.5	11	4.3	5.5	1.5
**DMFT**	**16**	**6.1**	**17**	**6.6**	**19**	**5.8**	**13.5**	**1.5**
**No. of decayed surfaces**	15	21.3	18	17.0	0.3	0.8	0	0
**No. of missing surfaces**	29	22.2	24	16.8	37	20.0	40	0
**No. of filled surfaces**	10.3	9.4	11	13.7	21	14.4	9	2
**DMFS**	**53**	**28.4**	**53**	**26.7**	**61**	**28.4**	**49**	**2**
**Plaque score** **No. of teeth with visible plaque)**	**54**	**23.2**	**N/A**	**N/A**	**39**	**15.2**	**N/A**	**N/A**

The BPE was reported as any one quadrant with BPE 3. In the MIOC group, five people didn't allow this measurement at the care planning session, so for 17 patients, the BPE was 3. At the follow up appointment, 6 people refused to have BPE measured and 10 people had 1 quadrant with BPE 3. In the control group (TAU) at the baseline 3 out of 7 people who allowed a measurement, had a BPE 3. At the follow up a participant had a BPE 3. Table 7 details the mean of OHIP-14 and TEI. The OHR QoL measured by OHIP-14 showed an improvement in both groups at the recall period. More people in the MIOC group accepted the provided services, however, it must be noted that the large number of dropouts in TAU makes a comparison difficult.

**Table 7:** The OHIP-14 and TEI scores for the study participants

	**MIOC baseline**		**TAU baseline**		**MIOC recall (18 people)**		**TAU recall (2 people)**	
	**Mean**	**Standard deviation**	**Mean**	**Standard deviation**	**Mean**	**Standard deviation**	**Mean**	**Standard deviation**
**Total OHIP-14 score**	**36**	**11.24**	**40**	**11.34**	**28**	**9.55**	**31.5**	**0.5**
**Total Adapted version of Treatment Evaluation Inventory (Patient)**	N/A	N/A	N/A	N/A	**49**	**5.18**	**41.5**	**1.5**

### Recall rates within the trial

In the MIOC (intervention arm) group, there were 4 dropouts whilst in the TAU group, the number was 19 (Fig. 1). There was an increased number of incomplete care / non-attendance in the TAU (control arm) group.

## Discussion

This feasibility study highlighted that a trial seeking to determine the effects of providing MIOC to patients with dental phobia is possible within a secondary care setting that is a major national provider of conscious sedation and CBT services. The recruitment process to identify eligible patients and acceptability of MIOC pathway by patients was successful as shown in the relatively low declining participating rates in the eligible participants (only 12% at the screening appointment and 17% at the care planning session). These factors indicate a possibility to conduct a future prospective MIOC randomised control trial (RCT) for people with dental phobia. The dentist fully adhered to the intervention (MIOC) protocol. The results from valid and reliable measures (MDAS, HADS, OHIP, caries diagnosis) also aided the comparison to other studies’ data.

The participants’ background (such as age, gender and educational levels) was similar to a published general population with phobia [20]. The study participants presented with better OHR behaviours (e.g. cleaning their teeth twice a day or more) than the general UK phobic population [2]. The participants’ self-reported OHR behaviours (e.g. use of electric toothbrush) further improved during the feasibility study with a high number of patients in the category of “patient behavioural adherence” in the MIOC group showed an improvement at the recall appointment.

This study’s high number of decayed teeth [2, 21–23] and the DMFT index (Armfield et al. 2009) have been comparable to other studies. At the recall period, there were improved clinical measures (the mean number of decayed teeth and plaques scores which had decreased while the number of filled had increased) in the same group (MIOC).

Previous studies [7, 24] have shown higher rates of scaling/polish and extractions with conscious sedation. The increased levels of plaque biofilm with a risk of caries associated with restorations, complexity of treatment for active unrestored large cavitated carious lesions (a complication of immediate or long-term biological complications in the "cycle of restoration" [25] and endodontic complications can be contributing factors to these performed treatments. The tooth retention rate can also dependent on dental anxiety levels (who might present challenges during treatment), tooth position [6] and access to dental care specifically with CS [7]. The increased number of missing teeth in the MIOC group can be a combination of the above mentioned factors.

Although the rate of the intervention (MIOC) groups’ follow up could be improved (a dropout rate of 4 people), the participants who completed the MIOC approach reported high satisfaction rates and expressed improved OHR QoL. The drop out rates in particular in TAU can partly been explained by participants’ phobic status (only people with MDAS scores of 19 and above were invited to take part). The lower drop out rate for the intervention arm may reflect the greater person-centred nature of the MIOC approach, especially if this was combined with CBT.

Although some anxious individuals report regular dental attendance [4, 26] many (like many participants in this study) only attend when having trouble with teeth [2]. Other explanatory factors can be people’s social demographic factors (e.g., poor educational background).

As a feasibility study, the population is a convenience sample with a fixed recall period that cannot give powerful statistical results [27]. There were many similarities (e.g., age, OHR behaviours) between the experimental (MIOC) and the control group (TAU) at the baseline which makes the recruitment to the study an easier process than anticipated and the interpretation of the data more acceptable. With no previously published studies about this patient group and MIOC, this feasibility has highlighted how a prospective RCT might be conducted and possible participants’ behaviour (e.g., individual tailored prevention regime’s impact on participants’ oral health outcomes in the MIOC group).

The lack of time was the reason for not collecting participants’ character and the reasons for refusals (rate of 48, [22%]), however, the refusal rate is comparable to a similar centre [28]. When looking at a previous SSCD study data, the study showed highly dentally anxious patients [11] who might have refused participation based on their phobic status.

The use of minimally invasive operative approaches (the third clinical domain of MIOC) to improve prognosis of treated teeth was welcomed by participants in this study unlike in the Schwendicke et al. (2016) [29] study. In their semi-structured focus group study, as part of their mixed-methods approach, Schwendicke and colleagues investigated treatment preferences (selective VS complete excavation) by using case-vignettes [29]. The regression analysis showed that people who showed higher dental anxiety and changed dentist more frequently were less reluctant to selective excavation. They argued that fear of recurrent caries, uncertainty and a need for re-treatment had an impact on their treatment preference.

This study bias was limited by using simple randomization with concealed allocation numbers and randomly reviewing researcher’s adherence to intervention. The introduced self-reported OHR behavioural changes can be a consequence of study participation, however, currently there are no evidence of a single effect [30] on participants’ reported behaviours. To minimise the Hawthorn effect, the researcher did not distribute or collect the anonymised questionnaires at the recall appointment.

A single examiner can be beneficial to provide a consistent care but there is a need to report intra-examiner reliability. The relatively short follow-up time might have overestimated the reported clinical effectiveness [31] with less decay teeth (mean 0.5, SD ± 1.4) and low restorations’ failure rate.

## Conclusion

With some uncertainties, a trial of MIOC for individuals with dental phobia is considered feasible and appropriate because people with dental phobia face multiple challenges from having access to care for their dental needs and dental phobia. Upon access, tackling their high levels of needs (anxiety and dental) has proven to be challenging. To address this, MIOC with its holistic approach and patient-focused care (where all team members can be involved) is suggested. The important components of MIOC, the prevention regime and MID data, did show a positive trend to deal with some of these encounters. MIOC and shared care seems to be a possibility for dental care provision in this group.

## Data Availability

All data generated or analysed during this study are included in this published article.
